# Conditional knockout of *Tsc1* in RORγt-expressing cells induces brain damage and early death in mice

**DOI:** 10.1186/s12974-021-02153-8

**Published:** 2021-05-06

**Authors:** Yafei Deng, Qinglan Yang, Yao Yang, Yana Li, Hongyan Peng, Shuting Wu, Shuju Zhang, Baige Yao, Shuhui Li, Yuan Gao, Xiaohui Li, Liping Li, Youcai Deng

**Affiliations:** 1 Hunan Children’s Research Institute (HCRI), Hunan Children’s Hospital, Changsha, 410000 China; 2 Institute of Materia Medica, College of Pharmacy, Army Medical University (Third Military Medical University), Chongqing, 400038 China; 3 Department of Pharmacy, The Third Xiangya Hospital, Central South University, Changsha, 410000 China; 4 Department of Clinical Biochemistry, Faculty of Pharmacy and Laboratory Medicine, Army Medical University (Third Military Medical University), Chongqing, 400038 China; 5 Southwest Hospital/Southwest Eye Hospital, Third Military Medical University, Chongqing, 400038 China

**Keywords:** *RORγt-*expressing cells, Tsc1, GABA, Seizure

## Abstract

**Background:**

Tuberous sclerosis complex 1 (Tsc1) is known to regulate the development and function of various cell types, and RORγt is a critical transcription factor in the immune system. However, whether *Tsc1* participates in regulating RORγt-expressing cells remains unknown.

**Methods:**

We generated a mouse model in which *Tsc1* was conditionally deleted from *RORγt-*expressing cells (*Tsc1*^*RORγt*^) to study the role of RORγt-expressing cells with *Tsc1* deficiency in brain homeostasis.

**Results:**

Type 3 innate lymphoid cells (ILC3s) in *Tsc1*^*RORγt*^ mice displayed normal development and function, and the mice showed normal Th17 cell differentiation. However, *Tsc1*^*RORγt*^ mice exhibited spontaneous tonic-clonic seizures and died between 4 and 6 weeks after birth. At the age of 4 weeks, mice in which *Tsc1* was specifically knocked out in *RORγt-*expressing cells had cortical neuron defects and hippocampal structural abnormalities. Notably, over-activation of neurons and astrogliosis were observed in the cortex and hippocampus of *Tsc1*^*RORγt*^ mice. Moreover, expression of the γ-amino butyric acid (GABA) receptor in the brains of *Tsc1*^*RORγt*^ mice was decreased, and GABA supplementation prolonged the lifespan of the mice to some extent. Further experiments revealed the presence of a group of rare *RORγt-*expressing cells with high metabolic activity in the mouse brain.

**Conclusions:**

Our study verifies the critical role of previously unnoticed RORγt-expressing cells in the brain and demonstrates that the Tsc1 signaling pathway in RORγt-expressing cells is important for maintaining brain homeostasis.

**Supplementary Information:**

The online version contains supplementary material available at 10.1186/s12974-021-02153-8.

## Introduction

Tuberous sclerosis complex 1 (Tsc1), which forms a heterodimeric complex with tuberous sclerosis complex 2 (Tsc2), controls and mediates major processes, including protein and lipid synthesis, autophagy, and cell survival and proliferation [1]. Disruption of the Tsc1-Tsc2 complex results in various human diseases, such as hamartomas in multiple organs, cortical dysplasia, hypomelanotic macules, and cancers [2, 3]. Previous studies found that targeted homozygous *Tsc1* or *Tsc2* mutations lead to death during the mid-embryonic period [4]. Mice with conditional *Tsc1* knockout in neurons or astrocytes exhibit abnormal brain structure and function [5]. For example, mice with neuronal *Tsc1* loss develop spontaneous seizures, macroencephaly, and hydrocephalus [6, 7]. Astrocyte-specific *Tsc1*-knockout mice also display epilepsy and some alterations in brain structure [8]. *Tsc1* also plays a critical role in regulating the development and functions of immune cells, such as T cells and NK cells. Deletion of *Tsc1* in CD4^+^ cells resulted in increased Th1 and Th17 cells differentiation [9], while conditional *Tsc1* deletion in Tregs impaired their suppressive activity [10]. Hematopoietic-specific deletion of *Tsc1* resulted in an activated and pro-apoptotic phenotype in NK cells [11]. However, the physiological role of *Tsc1*, especially in innate lymphoid cells (ILCs), remains largely unknown.

According to previous studies, RORγt is a critical transcription factor for type 3 innate lymphoid cells (ILC3s) and Th17 cells [12, 13]. RORγt deficiency leads to the failure of lymphoid tissue inducer cells (LTi cells) and failure to generate ILC3 subsets [14, 15]. Therefore, RORγt-Cre mice are now commonly used as an animal model to study the roles of specific genes in regulating ILC3s or Th17 cells. However, whether *Tsc1* participates in regulating RORγt-expressing cells remains unknown.

In this study, we found that mice with conditional Tsc1 deletion from RORγt-expressing cells (*Tsc1*^*RORγt*^ mice) had numbers of ILC3s in the intestinal lamina propria layer and Th17 cells in the spleen comparable to those of their control littermates. However, *Tsc1*^*RORγt*^ mice died in a narrow timeframe between 4 and 6 weeks after birth. As previous studies showed that Tsc1 deletion in neurons or astrocytes led to brain damage and death [6–8]; here, we further reveal that Tsc1^RORγt^ mice show obvious brain damage, including cortical neuron defects, hippocampal structural abnormalities, neuron over-activation, and astrogliosis in the cortex and hippocampus. Mechanistically, the loss of Tsc1 in RORγt-expressing cells resulted in lower γ-amino butyric acid (GABA) receptor expression in the brain, and GABA supplementation prolonged the lifespan of Tsc1^RORγt^ mice to some extent.

## Methods

### Animals

C57BL/6 *Rorc*-Cre (B6.FVB-Tg (*Rorc*-cre)1Litt/J, stock No. 022791) mice [16] and *Tsc1*^fl/fl^ (*Tsc1*^*tm1Djk*^/J, stock No. 005680) mice [17] were obtained from The Jackson Laboratory (Sacramento, CA, USA). All mice were bred under specific pathogen-free conditions at the Experimental Animal Center of Hunan Children’s Hospital (Changsha, China). For the GABA supplementation experiment, the *Tsc1*^*RORγt*^ mice and control littermates were fed GABA through the drinking water (0.5 mg/ml) at 3 weeks after weaning.

### Preparation of single-cell suspensions

Single-cell suspensions were prepared as previously described [18, 19]. Briefly, mice were anesthetized with an isoflurane vaporizer (4–5% v/v). Spleen tissues were ground and passed through a 70-μm stainless steel mesh, and pellets were collected after centrifugation (450×*g*, room temperature, 10 min). Spleen mononuclear cells were separated from the pellets by lysing erythrocytes. The small intestines were opened longitudinally and washed with PBS (pH 7.4, Sigma-Aldrich, St. Louis, MO, USA) to remove the contents after Peyer’s patches had been removed. Then, the intestines were cut into segments 4–5 cm in length and gently shaken in D-Hanks’ solution (pH 7.4) containing 10 mM HEPES, 5 mM EDTA, 1 mM DTT, and 10% fetal bovine serum (FBS) for 20 min at 37 °C. The remaining intestinal tissues were rinsed with Hanks’ solution and then digested with 1 mg/ml collagenase II (Gibco, Waltham, MA, USA) in Hanks’ solution (pH 7.4) supplemented with 10% FBS for 40 min at 37 °C with gentle shaking. The collected digests were filtered through a 100-μm mesh and enriched by centrifugation (450×*g*, room temperature, and 10 min) with a 25% Percoll solution (GE Healthcare, Pittsburgh, PA, USA) in RPMI 1640 medium (Gibco).

### Antibodies and flow cytometry

All antibodies used for staining and subsequent flow cytometry analysis are listed in Supplemental Table 1. We confirmed the species reactivity for all antibodies according to the manufacturer’s directions and performed preliminary experiments to determine the appropriate dilutions for all antibodies. Standard protocols were used for flow cytometry [19, 20]. Briefly, single-cell suspensions were obtained from the spleen and intestinal lamina propria tissues of the mice. To analyze surface markers, 2 × 10^6^ cells were incubated with staining buffer (PBS containing 2% mouse serum, 2% horse serum, and anti-CD16/CD32 blocking antibodies (BioLegend, San Diego, CA, USA)) and the indicated surface antibodies for 15 min at room temperature.

To detect IL-17A and IL-22, 2 × 10^6^ cells were stimulated with IL-23 (BioLegend) or PMA/ionomycin (BD Biosciences, San Diego, CA, USA) for 4 h in the presence of BD Golgi Plug™ protein transport inhibitor (BD Biosciences) at 37 °C. Then, the cells were fixed with a Fixation/Permeabilization Solution Kit (BD Biosciences) according to the manufacturer’s instructions.

For RORγt staining, cells were stained with antibodies against surface markers, permeabilized with the Foxp3/Transcription Factor Staining Buffer Set Kit (eBioscience, San Diego, CA, USA), and then stained with anti-RORγt antibody. Lineage (Lin) markers included CD3e, CD19, B220 and Gr-1. All of the isotype-matched control antibodies were purchased from BioLegend and BD and used at the same concentration as the test antibodies. All flow cytometry experiments were performed with a BD FACSCanto™ or BD FACS LSRFortessa™ instrument (BD Biosciences). Data were analyzed with FlowJo software (version 10.0, FlowJo LLC, Ashland, OR, USA). The lines indicate median values for each group.

### Histological analysis

The mice were anaesthetized with an isoflurane vaporizer (4–5% v/v), and all tissues were collected as described in our previous reports [18, 21]. Histological structures of the heart, lung, kidney, spleen, stomach, liver, and brain were analyzed by standard hematoxylin-eosin (HE) staining and assessed for the presence of pathomorphological changes by trained pathologists who were blinded to the experimental groups. The Nissl bodies in the brain were detected by Nissl staining. Briefly, the tissues were fixed in 10% neutral buffered formalin for at least 24 h, embedded in paraffin, and cut into 4-μm-thick sections. The paraffin sections were stained with HE or Nissl staining solutions after they had been processed according to standard protocols [22].

All antibodies used for immunofluorescence staining are listed in Supplemental Table 1. The brain sections were incubated with a polyclonal rat anti-mouse glial fibrillary acidic protein (GFAP) antibody (eBioscience) and/or polyclonal rabbit anti-mouse neuronal nuclei antigen (NeuN) antibody (eBioscience) in 10% goat serum overnight at 4 °C after deparaffinization, rehydration, and antigen retrieval. The sections were washed with 1× PBST (1× PBS, 0.05% Tween-20), incubated with secondary antibodies (Alexa Fluor® 488-conjugated goat anti-rat IgG (H+L) for GFAP and Alexa Fluor® 594-conjugated goat anti-rabbit IgG (H+L) for NeuN) for 1 h at room temperature and then stained with DAPI. Coverslips containing samples from all organs, except for the brain, subjected to HE staining were scanned using an Olympus VS200 slide scanner (Olympus, Japan) and visualized with OlyVIA version 3.2 software (Olympus). Coverslips containing brain samples were scanned using Pannoramic DESK (3D HISTECH, Budapest, Hungary) and visualized with CaseViewer software (3D HISTECH). Measurements of cortical thickness and cell scattering width in the CA1, CA3, and dentate gyrus (DG) regions of the hippocampus were performed using CaseViewer software (3D HISTECH). Quantitative analyses of the area fraction of GFAP^+^ astrocytes, NeuN^+^ neurons, and Nissl bodies were performed using ImageJ software version 1.52v (National Institutes of Health, Bethesda, MD, USA) [23]. The investigators who acquired and analyzed the images were blinded to the experimental groups.

### Electrocardiogram measurement

The mice were anaesthetized with an isoflurane vaporizer (4–5% v/v), and the electrocardiogram (ECG) was measured and recorded by electrocardiography (Mindray, Shenzhen, China) according to the operation manual.

### Detection of mRNA levels by real-time RT-PCR

Briefly, mice were anesthetized with an isoflurane vaporizer (4–5% v/v), and whole-brain parenchyma and thymus tissues then were harvested. Total RNA was extracted and reverse transcribed into cDNA using standard protocols as previously described [24]. Total RNA was extracted from the brain and thymus tissues using TRIzol reagent (Invitrogen, Waltham, MA, USA), and cDNA was synthesized using a First Stand cDNA Synthesis Kit (DBI Bioscience, Ludwigshafen, Germany). Real-time PCR was performed using Bestar® SYBR Green qPCR Master Mix (DBI Bioscience, San Diego, CA, USA). The cycle threshold (Ct) values were normalized to the internal control (*gapdh*). The primer sequences for qRT-PCR were obtained from the Primer Bank, and the primer pairs used in the present study are shown in Supplemental Table 2.

### Western blotting

Briefly, mice were anesthetized with an isoflurane vaporizer (4–5% v/v). Then, whole-brain parenchyma and thymus tissues were harvested and lysed with RIPA buffer (Sigma-Aldrich), separated by SDS-PAGE, transferred to PVDF membranes (GE Healthcare, Pittsburgh, PA, USA), and incubated with anti-RORγt antibody in 5% BSA [23]. Immunoblotting was performed using HRP-conjugated secondary antibodies for visualization.

### Seizure test

Seizures were induced with pentylenetetrazole (PTZ) as previously described [25]. PTZ (50 mg/kg, Sigma-Aldrich) was administered intraperitoneally at 10-min intervals to post-natal days (PNDs) 28 mice. The injections were administered until the mice died (1–3 injections). Seizure latency was recorded as the time from PTZ injection to seizure occurrence. Observers were blinded to the genotypes of the mice.

### Single-cell data analysis

The raw gene count expression matrix and metadata from embryonic day 14.5 mouse cerebral cortex scRNA-seq datasets were downloaded from GEO (https://www.ncbi.nlm.nih.gov/geo/) (accession ID: GSE123335) [26] for analysis. Dimensional reduction, clustering, and analysis of scRNA-seq data were performed with the R package Seurat version 3 [27]. Cells with a RORC gene expression level ≥ 1 were considered RORγt-positive cells. The top 25% of the median of all expressed genes in RORγt-positive cells were considered highly expressed genes and used for KEGG enrichment analysis based on ClueGO to analyze the potential functions of RORγt-positive cells [28]. Gene set variation analysis (GSVA), a nonparametric and unsupervised software algorithm, was used to assess KEGG pathway activation with the R package GSVA [29].

### Statistical analyses

All quantitative data were transferred to Excel, and statistical analyses were carried out with SPSS software for Windows (version 21, SPSS Inc., Chicago, Illinois, USA). Data are presented as the means ± SEMs. For comparisons between two independent experimental groups, an unpaired two-tailed Student’s *t* test was used when data were normally distributed. When more than two independent groups were compared, one-way ANOVA followed by Tukey’s test was performed. The Wilcoxon test was used to compare the difference in KEGG pathway scores estimated by GSVA between *RORγt-*positive and *RORγt-*negative cells with R (version 3.5.1) [30]. A *p* value less than 0.05 was considered to indicate statistical significance. Each analysis included *n* = 4–18 replicates per group, and the results are representative of at least two independent experiments. The sample size for each experiment is indicated in the corresponding figure legend. All graphs were produced using GraphPad Prism 5.0 for Windows (GraphPad Software Inc., La Jolla, CA, USA).

## Results

### Deletion of *Tsc1* in *RORγt-*expressing cells resulted in the unexpected death of mice

**Fig. 1:**
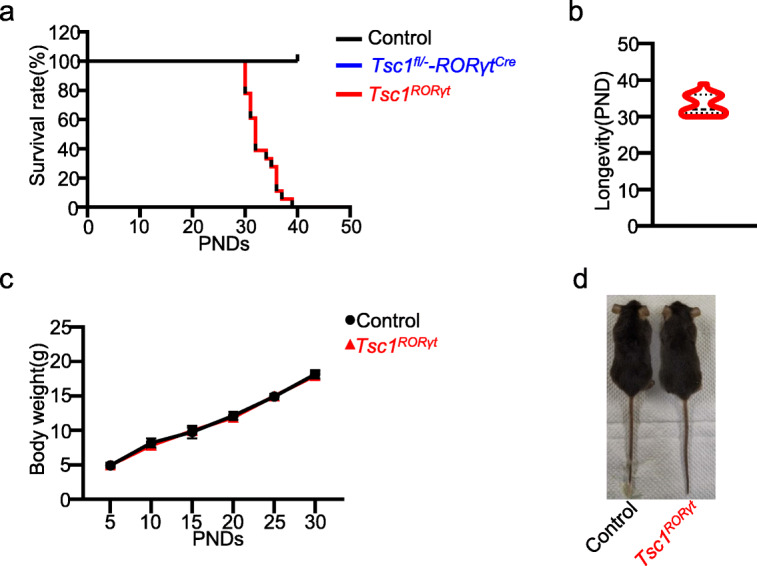
The deletion of *Tsc1* in *RORγt-*expressing cells resulted in the unexpected death of mice. **a**–**d** The survival rate (**a**), longevity (**b**), body weight (**c**), and appearance (**d**) of *Tsc1*^RORγt^ mice and control littermates. One-way ANOVA (**a**). *n* = 9–18 (**a**), *n* = 18 (**b**), *n* = 6 (**c**). Unpaired two-tailed Student’s t test (c).

To investigate whether *Tsc1* deletion would affect *RORγt-*expressing cells, we crossed *Tsc1* floxed (*Tsc1*^fl/fl^) mice with *RORγt*-Cre mice to obtain *Tsc1*^fl/fl^-*RORγt*^Cre+^ mutant mice in which Tsc1 was deleted from only *RORγt-*expressing cells (referred to as *Tsc1*^*RORγt*^ mice), while *Tsc1*^fl/fl^-*RORγt*^Cre-^ littermates were used as control mice. We initially aimed to explore the effects of *Tsc1* deficiency on ILC3s and Th17 cells; however, all *Tsc1*^*RORγt*^ mice unexpectedly died between PNDs 30 and PNDs 40, with a median survival time of 33 days (Fig. 1a, b). However, mice with heterozygous *Tsc1* knockout in *RORγt-*expressing cells (*Tsc1*^fl/-^-*RORγt*^Cre^) had a lifespan similar to that of control littermates (Fig. 1a). The *Tsc1*^*RORγt*^ mice had a normal weight and did not display gross morphological abnormalities upon comparison with control littermates before their death (Fig. 1c, d).

### ILC3s and Th17 cells in *Tsc1*-deficient *RORγt-*expressing cells showed normal phenotypes

**Fig. 2:**
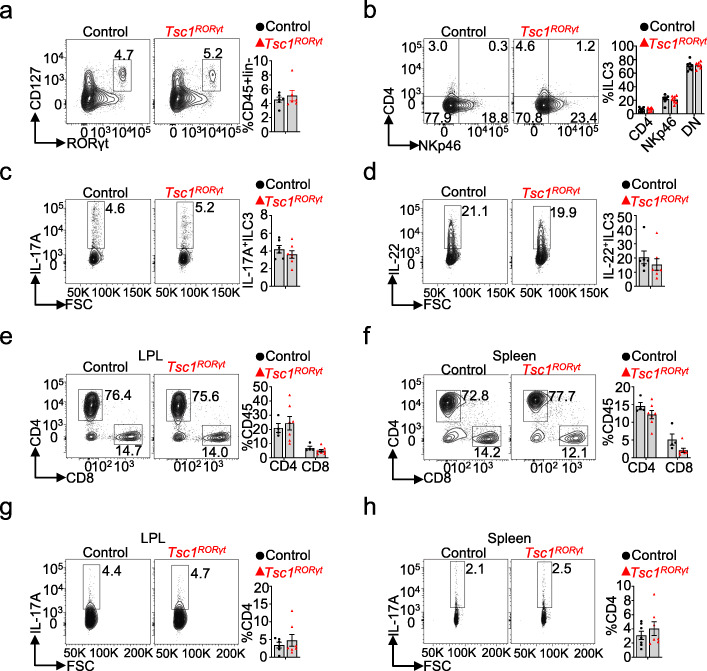
ILC3s and Th17 cells from *Tsc1*-deficient *RORγt-*expressing cells showed normal phenotypes. **a**, **b** Flow cytometry analysis and cumulative frequencies of total ILC3s (**a**) and their subsets (**b**) in the LPL of *Tsc1*^RORγt^ mice. **c**, **d** Flow cytometry analysis and cumulative frequencies of IL-17-producing and IL-22-producing ILC3s in the small intestine LPL of *Tsc1*^RORγt^ mice. **e**, **f** Flow cytometry analysis and cumulative frequencies of CD4^+^ T cells and CD8^+^ T cells in the LPL (**e**) and spleen (**f**), respectively. **g**, **h** Flow cytometry analysis and cumulative frequencies of IL-17-producing CD4^+^ T cells in the LPL (**g**) and spleen (**h**), respectively. Each dot represents one mouse; error bars represent SEMs. Unpaired two-tailed Student’s *t* test (**a–h**)

First, we postulated that the early death of the mice had been induced by the knockout of *Tsc1* in ILC3s or Th17 cells, as according to the description of mice homozygous for the *RORC* mutant allele (RORγt^GFP/GFP^) from The Jackson Laboratory, ROR*γt*-deficient mice were reported to die at the age of 9–32 weeks [16]. Because these *Tsc1*^*RORγt*^ mice died between PNDs 30 and PNDs 40, all analyses of the mice were conducted at PNDs 28 unless specified otherwise. Flow cytometry analysis showed that *Tsc1*^*RORγt*^ mice had numbers of ILC3s and ILC3 subsets comparable to those of their control littermates (Fig. 2a, b). Moreover, *Tsc1* deficiency in RORγt-expressing cells did not affect the expression of IL-17A or IL-22 (Fig. 2c, d), the main cytokines expressed in ILC3s, in this cell type [31]. As *RORγt* is also expressed in Th17 cells, we determined the ratio of Th17 cells in these mice and found that ratios of CD4^+^ T cells, CD8^+^ T cells, and Th17 cells in the intestinal lamina propria layer (LPL) and spleen were comparable in *Tsc1*^*RORγt*^ mice and their control littermates (Fig. 2e–h).

**Fig. 3:**
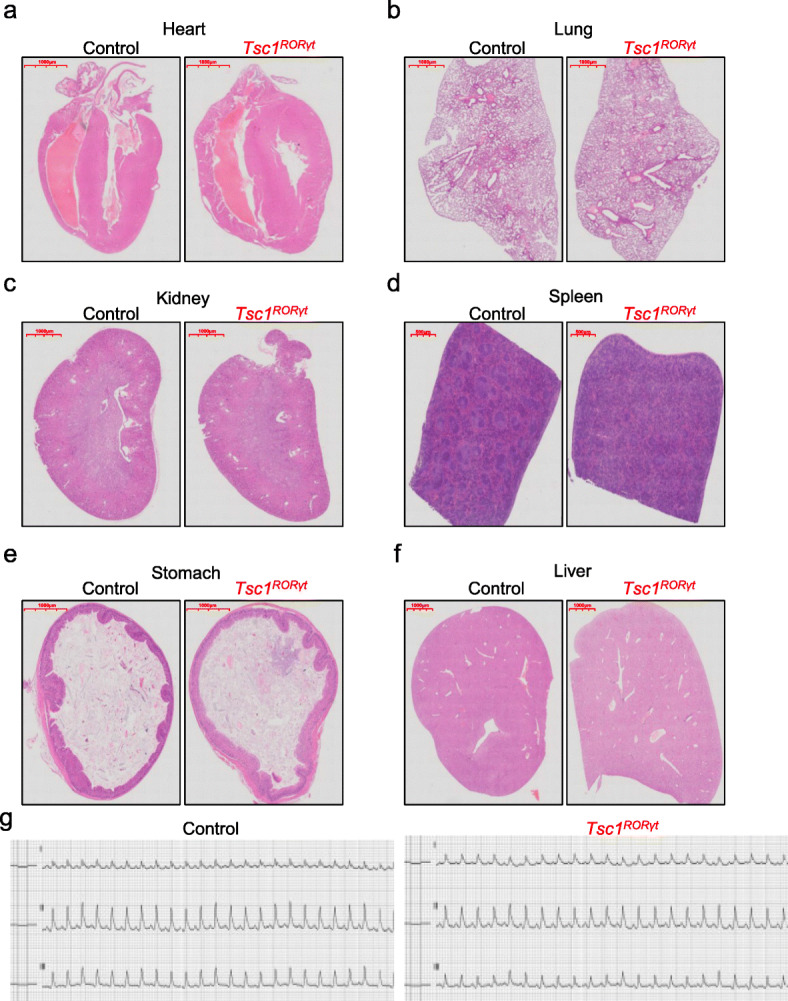
*Tsc1*^*RORγt*^ mice did not display significant pathomorphological changes in various tissues and exhibited normal cardiac function. **a** HE staining and representative images of the heart, lung, kidney, spleen, stomach, and liver of *Tsc1*^RORγt^ mice and control littermates. **b** Representative ECG measurements from *Tsc1*^RORγt^ mice and control littermates. *n* = 5 animals per group. All HE staining images were captured from scans of whole-tissue slices. Scale bars: 500 μm (**d**) and 1000 μm (**a**, **b**, **c**, **e**, **f**)

To further investigate the cause of death, we assessed pathological changes in several critical organs, including the heart, lung, kidney, spleen, stomach, and liver, of *Tsc1*^*RORγt*^ mice by hematoxylin-eosin (HE) staining. *Tsc1*^*RORγt*^ mice did not display significant pathomorphological changes that would have led to their death (Fig. 3a–f). Meanwhile, ECG measurements also revealed that *Tsc1*^*RORγt*^ mice had normal cardiac function (Fig. 3g). These results strongly suggest that the death of the mice caused by *Tsc1* deficiency in *RORγt*-expressing cells was independent of ILC3s and Th17 cells.

### *Tsc1*^*RORγt*^ mice exhibit spontaneous tonic-clonic seizures with neuronal defects

**Fig. 4:**
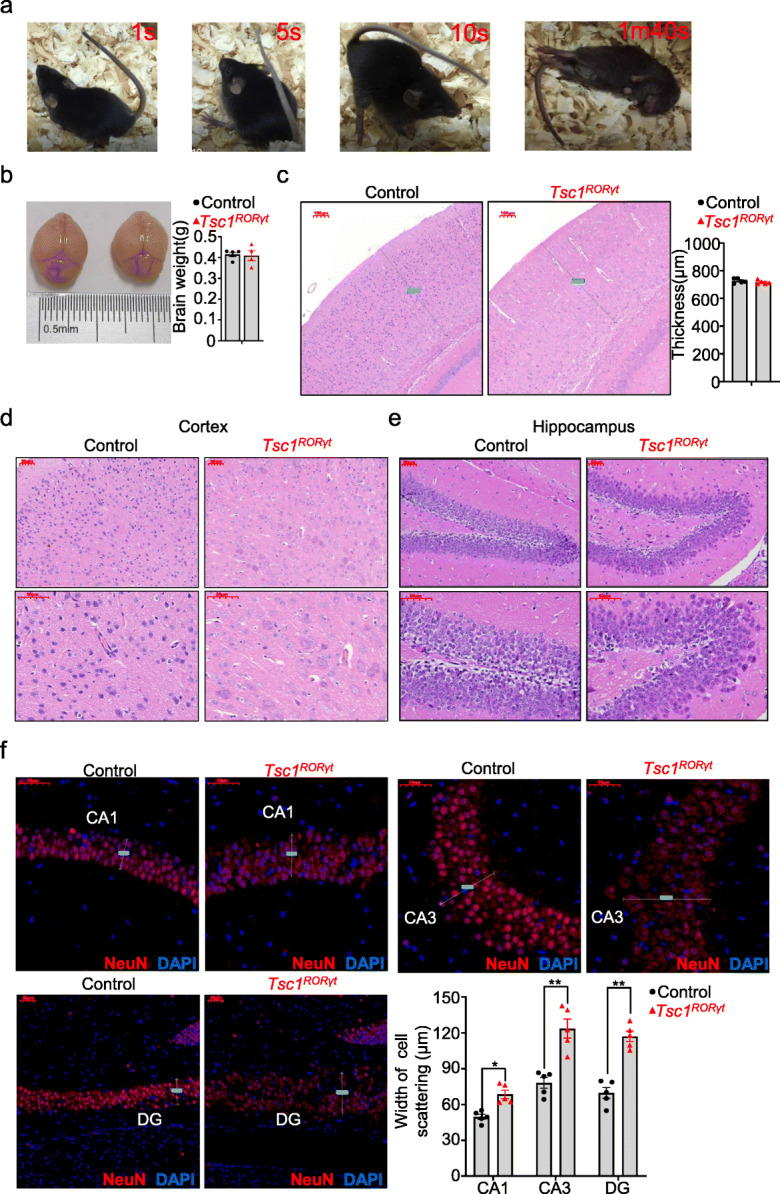
*Tsc1*^*RORγt*^ mice exhibited spontaneous tonic-clonic seizures with neuronal defects. **a** Representative images of spontaneous generalized tonic-clonic seizures at different phases observed in the *Tsc1*^RORγt^ mice. These images were extracted from a video of *Tsc1*^RORγt^ mice housed with control littermates. **b** The brain weights of *Tsc1*^RORγt^ mice and control littermates. **c** Representative images of HE staining and thickness of the cortex of the brains of *Tsc1*^RORγt^ mice and control littermates. **d, e** Representative HE staining images of the cortex (**d**) and hippocampus (**e**) of *Tsc1*^RORγt^ mice and control littermates. **f** Representative images of NeuN immunostaining (red) and the cell scattering width in the CA and DG regions of the hippocampus from *Tsc1*^RORγt^ mice and control littermates. Each dot represents one mouse, and error bars represent SEMs; *p < 0.05, **p < 0.01. Unpaired two-tailed Student’s *t* test (**b**, **c**, **f**). All images were captured from scans of whole-brain slices. Magnification: × 10 (**c**), × 20 (upper panels in **d**, **e**), and × 40 (lower panels in **d, e**, **f**). Scale bars: 50 μm (**d**, **e**, **f**) and 100 μm (**c**)

Unexpectedly, the *Tsc1*^*RORγt*^ mice often suffered megascopic spontaneous seizures characterized by generalized tonic-clonic activity before PNDs 28 (Supplemental Video 1). The seizure activity became more severe with increasing age, ultimately resulting in wild jumping and death (Fig. 4a and Supplemental Video 2). We next assessed pathomorphological changes in the brain to further investigate the cause of death in the *T*s*c1*^*RORγt*^ mice. The brain size and cerebral cortical thickness of the Ts*c1*^*RORγt*^ mice were the same as those of control littermates (Fig. 4b, c). However, neurons in the cerebral cortex of Ts*c1*^*RORγt*^ mice appeared swollen and necrotic, unlike those of control littermates (Fig. 4d). The hippocampus of Ts*c1*^*RORγt*^ mice also exhibited defects in organization. HE staining showed that structure of the dentate gyrus (DG) in the hippocampus was disorganized, and the arrangement of neurons was also disordered in the Ts*c1*^*RORγt*^ mice (Fig. 4e). Immunofluorescence staining for NeuN showed that the CA1 and CA3 pyramidal cell layers and DG granule cell layer were organized in an irregular fashion and that cells in these regions were discrete in Ts*c1*^*RORγt*^ mice. Quantitative analysis revealed increased cell scattering widths in the CA1, CA3, and DG cell layers of Ts*c1*^*RORγt*^ mice (Fig. 4f). These results suggest that the loss of *Tsc1* in *RORγt*-expressing cells resulted in neuronal perturbations in the cortex and structural abnormalities in the hippocampus.

### Abnormal activation of neurons and astrogliosis in the cortex and hippocampus of Ts*c1*^*RORγt*^ mice

**Fig. 5:**
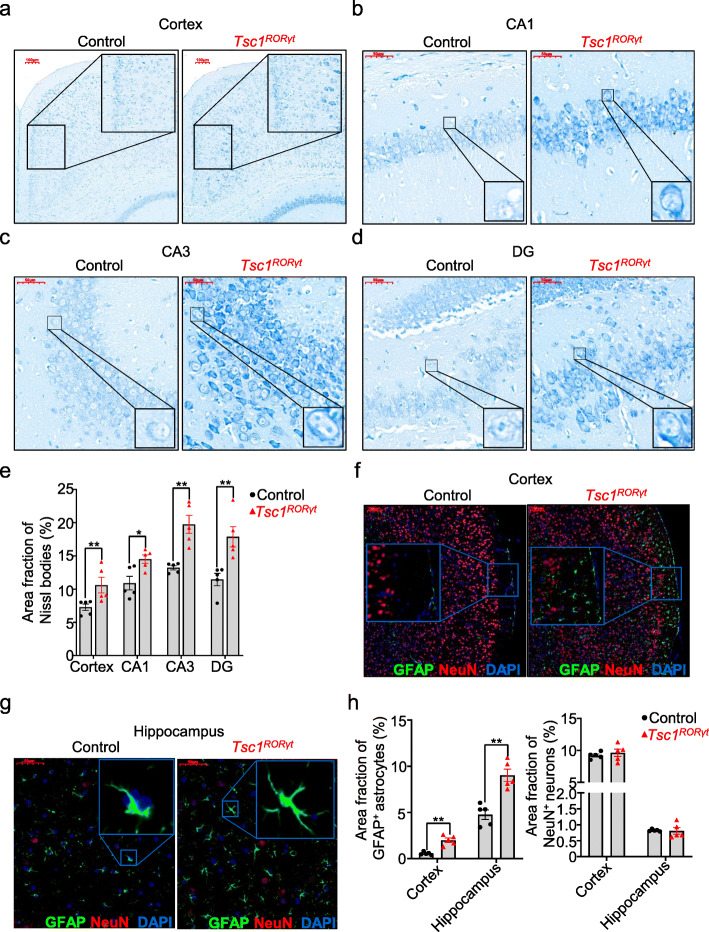
Abnormal activation of neurons and astrogliosis in the cortex and hippocampus of Ts*c1*^*RORγt*^ mice. **a**–**d** Representative images of Nissl staining of the cerebral cortex (**a**) and different regions of the hippocampus (**b**–**d**) of *Tsc1*^RORγt^ mice and control littermates. **e** Statistical analysis of the area fraction of Nissl bodies shown in **a**–**d**. **f**–**h** Representative images of GFAP immunostaining (green) (**f** and **j**) and the area fraction of GFAP^+^ astrocytes and NeuN^+^ neurons (**h**) in the cerebral cortex (**f**) and hippocampus (b) of *Tsc1*^RORγt^ mice and control littermates. Each dot represents one mouse and the error bars represent SEMs; *p < 0.05 and **p < 0.01. Unpaired two-tailed Student’s *t* test (**e**, **f**, **g**). The locations of the magnified box are identified in the original image. All images were captured from scans of whole-brain slices. Magnification: 10x (**a**, **f**) and × 40 (**b**, **c**, **d**, **g**). Scale bars: 50 μm (**b**, **c**, **d**, **g**) and 100 μm (**a**, **f**)

Seizures are usually characterized by the abnormal and excessive synchronous firing of neurons [32]. Nissl bodies are usually regarded as an indicator of neuronal activation, in the brain [33]; therefore, we determined the number of Nissl bodies in CA1 and CA3 pyramidal cell layers and the DG granule cell layer of both control and Ts*c1*^*RORγt*^ mice. Compared with control littermates, Ts*c1*^*RORγt*^ mice exhibited an increased number of Nissl bodies with a complex microstructure, as evidenced by deeper staining and an increased area fraction of Nissl bodies in the cortex and hippocampus, indicating that Ts*c1*^*RORγt*^ mice exhibited abnormal neuronal activation (Supplemental Fig. 1a and Fig. 5a–e). However, the deletion of Tsc1 in RORγt-expressing cells did not affect the number of total neurons in the CA1 or CA3 pyramidal cell layer or the DG granule cell layer, as indicated by quantitative analysis of the area fraction of NeuN^+^ neurons (Supplemental Fig. 1b and Fig. 5f–h).

Astrogliosis, which refers to an abnormal increase in astrocytes, usually occurs when an insult to the brain is sustained and often presents in patients with tuberous sclerosis syndrome or epilepsy [34]; elevated GFAP expression is a marker of astrogliosis [35]. We observed weakly stained astrocytes in the cerebral cortex of control mice; moreover, the area fraction of GFAP^+^ astrocytes was obviously increased in the cortex of *Tsc1*^RORγt^ mice compared to control littermates (Fig. 5f, h). Moreover, compared with that of control littermates, increased GFAP expression was also found throughout the hippocampus of *Tsc1*^RORγt^ mice, in which GFAP^+^ astrocytes were widely distributed, especially in the DG region (Supplemental Fig. 1b and Fig. 5g, h).

Thus, deletion of *Tsc1* from RORγt-expressing cells did not affect the ratios or phenotypes of ILC3s or Th17 cells but resulted in abnormal activation of neurons and astrogliosis, which might have been responsible for the seizures observed in the *Tsc1*^RORγt^ mice.

### Defects in the GABA signaling pathway are at least partially responsible for seizures and death in *Tsc1*^*RORγt*^ mice

**Fig. 6:**
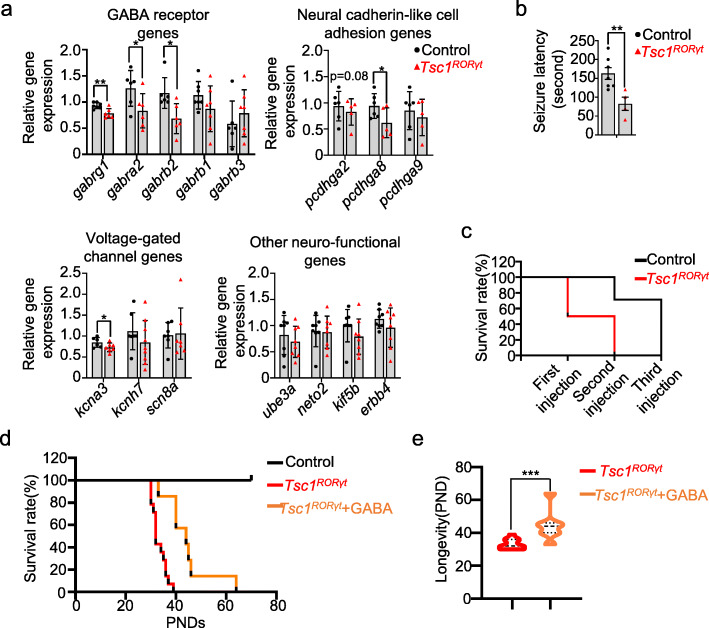
Defects in the GABA signalling pathway are at least partially responsible for seizures and death in *Tsc1*^*RORγt*^ mice. **a** Relative mRNA expression of GABA receptor subunit genes, neural cadherin-like cell adhesion genes, voltage-gated channel genes, and other genes related to neuronal function in the whole brains of *Tsc1*^RORγt^ mice and control littermates. **b**, **c** Seizure latency (**b**) and survival rate (**c**) of *Tsc1*^RORγt^ mice and control littermates after PTZ administration. **d**, **e** Survival rate (**d**) and longevity (**e**) of *Tsc1*^RORγt^ mice, *Tsc1*^RORγt^ mice supplemented with GABA and control littermates. Each dot represents one mouse, and error bars represent SEMs; *p < 0.05, **p < 0.01, and ***p < 0.001. *n* = 4–7 (**c**) and *n* = 7–14 (**d**, **e**). Unpaired two-tailed Student’s *t* test (**a–c**, **e**). One-way ANOVA (**d**)

To further investigate the underlying mechanisms of these abnormalities in the nervous system, we measured the gene expression levels of GABA receptor subunit genes (*gabrg1*, *gabra2*, *gabrb2*, *gabrb1*, and *gabrb3*), neural cadherin-like cell adhesion genes (*pcdhga2*, *pcdhga8*, and *pcdhga9*), voltage-gated channel subunits (*kcnh7*, *kcna3*, and *scn8a*), and other genes related to neuronal function (*ube3a*, *neto2*, *kif5b*, and *erbb4*) [36, 37] in the whole brains of both *Tsc1*^RORγt^ and control littermate mice by quantitative real-time RT-PCR. The GABA signaling pathway is the major inhibitory neural pathway and mediates slow and prolonged inhibitory activity [36], and defects in the GABA signaling pathway increase seizure susceptibility [38]. *Pcdhg* genes regulate neuronal survival, cortical interneuron programmed cell death, and neural circuit assembly [39, 40]. Sodium and potassium voltage-gated channel genes are also associated with neurological disorders [41–43]. Additionally, other genes have been reported to regulate neuronal function [44–47]. Notably, the expression levels of *gabrg1*, *gabra2*, and *gabrb2* were significantly downregulated in the brains of *Tsc1*^RORγt^ mice compared with control littermates. Additionally, *pcdhga2* and *pcdhga8* and *kcna3* levels were decreased in the brains of *Tsc1*^RORγt^ mice (Fig. 6a).

To determine whether defects in the GABA signaling pathway resulted in seizures and death in the Ts*c1*^*RORγt*^ mice, the mice were injected with increasing doses of PTZ (a GABA receptor antagonist, 50 mg/kg, *i.p*.) [25], and the subsequent latency of generalized tonic-clonic seizures was determined. Compared with control littermates, Ts*c1*^*RORγt*^ mice showed a significant reduction in seizure latency (162.8 ± 41.5 s vs. 82.5 ± 35.0 s, respectively) after PTZ challenge (Fig. 6b). In addition, Ts*c1*^*RORγt*^ mice exhibited a 50% mortality rate after the first PTZ injection and 100% mortality rate after a second PTZ injection, while control littermates began to die with a 30% mortality rate after the second PTZ injection, and all died after the third PTZ injection (Fig. 6c).

To confirm the role of deficiency in the GABA signaling pathway in the death of Ts*c1*^*RORγt*^ mice, the mice were fed GABA through the drinking water starting at the age of 3 weeks after weaning. Although GABA supplementation did not prevent the death of the *Tsc1*^RORγt^ mice, their survival time was prolonged, with a median value of 44 days (Fig. 6d, e). These data reveal that *Tsc1* deficiency in RORγt-expressing cells led to defects in the GABA signaling pathway that at least partially contributed to seizures and death in *Tsc1*^RORγt^ mice.

### Presence of a group of rare RORγt-positive cells with a high metabolic level in the mouse brain

**Fig. 7:**
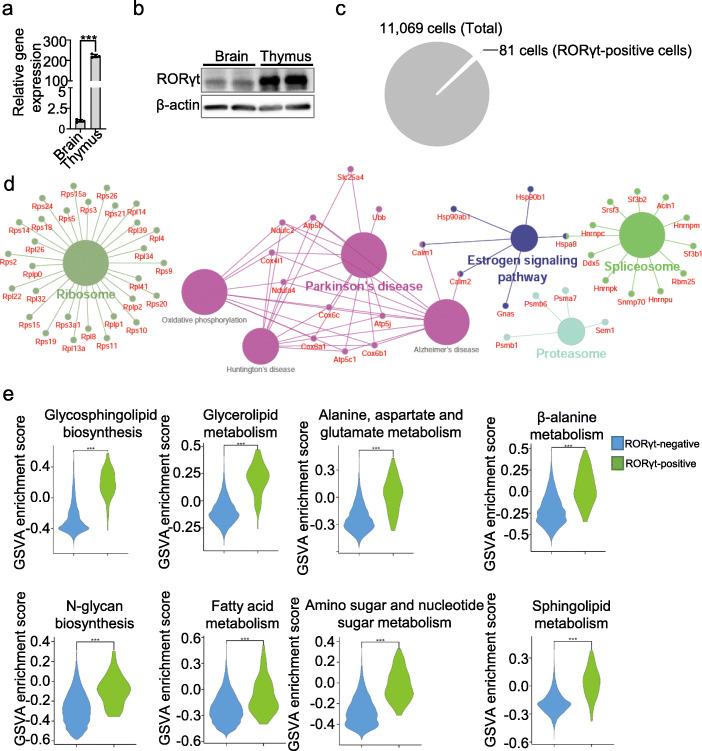
A group of rare RORγt-positive cells with a high metabolic level exists in the mouse brain. **a**, **b** Relative expression of the *RORγt* mRNA in the whole brain and thymus (**a**) and *RORγt* protein expression in the whole brains (**b**) of mice at PNDs 14. **c** Proportion of *RORγt-*positive cells among total brain cells. **d** Functional enrichment analysis of the top 25% most highly expressed genes in *RORγt-*positive cells. **e** Functional gene sets identified by KEGG pathway enrichment analysis indicated increased metabolic pathway enrichment in *RORγt-*positive cells compared with *RORγt-*negative cells. Each dot represents one mouse, and error bars represent SEMs; ***p < 0.001. Unpaired two-tailed Student’s *t* test (**a**). Wilcoxon test (**e**)

The deletion of *Tsc1* in RORγt-expressing cells did not affect ILC3s or Th17 cells; therefore, we speculated that a group of *RORγt*-expressing cells may be present in the brain and that the deletion of *Tsc1* in these cells would induce brain dysfunction, resulting in seizures, and eventual death. Therefore, we first analyzed *RORγt* expression in the whole brain by using data from the Allen Brain Atlas (http://mouse.brain-map.org/). Indeed, in situ hybridization (ISH) analysis indicated that *RORγt* was expressed in the cortex of the cerebrum and cerebellum during the embryonic period (E18.5). After birth, its expression increased and peaked at PNDs 14 and then decreased at PNDs 28 (Supplemental Fig. 2). Moreover, our RT-PCR and Western blotting data showed a very low level of RORγt expression in the brain that is much lower than its expression in the thymus (Fig. 7a, b). Therefore, we confirmed that, although rare, RORγt-positive cells are present in the brain.

By re-analyzing published single-cell RNA-seq data from the mouse brain [26], we identified 81 RORγt-positive cells among 11,069 cells (0.73% of total cells) (Fig. 7c). Functional enrichment analysis revealed that these RORγt-positive cells exhibited high expression levels of ribosomal and spliceosome-associated genes. Many of the highly expressed genes in RORγt-positive cells are also involved in both oxidative phosphorylation and neurological disorders, including Parkinson’s disease, Huntington’s disease, and Alzheimer’s disease (Fig. 7d). Meanwhile, KEGG enrichment analysis indicated that 139 signaling pathways were significantly differently enriched between RORγt-positive cells and RORγt-negative cells (Supplemental Table 3). Among these pathways, many pathways related to metabolism, including glycosphingolipid biosynthesis and amino acid and fatty acid metabolism, were enriched in RORγt-positive cells, indicating that the metabolic level in RORγt-positive cells is enhanced compared to that of RORγt-negative cells in the brain (Fig. 7e). These data suggest the presence of a group of rare RORγt-positive cells with a high metabolic level in the mouse brain.

## Discussion

Our study shows that *Tsc1* expression in RORγt-expressing cells is dispensable for ILC3 development and function but critical for brain homeostasis. Loss of *Tsc1* in RORγt-expressing cells in the mouse brain induced neuronal defects, astrogliosis in the cortex and hippocampus, spontaneous tonic-clonic seizures, and death. Notably, GABA supplementation delayed death and prolonged lifespan to some extent.

According to previous studies, Th17 cells can infiltrate the brains of patients of Parkinson’s disease and induce neuronal cell death [48], and *Tsc1* deficiency affects T cell development and function [9, 10]. However, our data showed that knockout of *Tsc1* in RORγt-expressing cells did not affect the development of Th17 cells; however, *Tsc1* knockout caused severe brain damage followed by death at the age of 4 to 6 weeks. Previous studies reported that neuron-specific inactivation of *Tsc1* resulted in the increased generation of neural progeny and death with a median survival time of 18 days [7], while the specific inactivation of *Tsc1* in GFAP-positive astrocytes led to astrogliosis and death between 11 and 22 weeks after birth [8]. These mice with astrocyte- and neuron-specific inactivation of *Tsc1* had an enlarged brain and edema. However, the brains of our model *Tsc1*^RORγt^ mice showed a normal appearance. The loss of *Tsc1* in RORγt-expressing cells not only caused neuronal defects but also induced astrogliosis, indicating that epilepsy and death caused by the inactivation of *Tsc1* in RORγt-expressing cells might be due to both the abnormal discharge of neurons and activated astrocytes. Because *RORγt* is expressed in the brain during the embryonic period (E18.5), the *Tsc1* gene was knocked out in *RORγt*-expressing cells in utero. Based on the findings, abnormal brain structures might appear during embryonic development, and a vicious circle between a disordered brain structure and seizures further aggravates brain damage with increasing age.

GABA, the major neurotransmitter in the central nervous system, is an important regulator of neuronal inhibition as its binds GABA_A_ receptors, which are ligand-gated anion channels. Dysfunction or mutation of GABA_A_ receptors has been identified in patients with various types of epilepsy [49]. *Tsc1*^RORγt^ mice exhibited a decreased latency to PTZ-induced seizures and were more susceptible to PTZ than control littermates, potentially due to decreased expression of GABA_A_ receptor subunits in the brain. Moreover, GABA supplementation prolonged the lifespan of *Tsc1*^RORγt^ mice, which further suggests that the impairment of GABA-GABA_A_ receptor interactions exacerbated seizures. Meanwhile, the loss of *Tsc1* in RORγt-expressing cells also decreased the expression levels of neuronal function-related genes (*pcdhga2* and *pcdhga8*) and ion channel gene expression (*kcna3*). *Pcdhg* genes were reported to regulate neuronal survival, synaptic maintenance, and neural circuit assembly [40, 50], which might explain why GABA supplementation alone did not improve the survival rate of *Tsc1*^RORγt^ mice. The *Kcna3* gene encodes the voltage-gated potassium channel Kv1.3, which protects against neuro-inflammation [43, 51–53]. Thus, we proposed that the decreased expression of *kcna3* was a compensatory mechanism when neuronal defects and astrogliosis occurred in the brains of *Tsc1*^RORγt^ mice.

According to the Allen Brain Atlas, RORγt is expressed in the brain during the embryonic period, and its expression increases after birth. In our study, the expression of RORγt in the brain parenchyma was confirmed by RT-PCR and Western blot analysis and then verified by analysis of published single-cell RNA-seq data. Interestingly, these rare RORγt-positive cells were found to be critical for brain homeostasis. Compared with RORγt-negative cells, RORγt-positive cells exhibited an increased metabolic level, as evidenced by higher levels of ribosomal gene expression and oxidative phosphorylation. Oxidative phosphorylation is an essential process for cell function, and ribosomes are targeted for autophagy-mediated degradation to provide supplemental amino acids and nucleotides under conditions of nutrient starvation [54].

A recent study identified a cluster of RORγt-positive γδ T cells in the meninges that regulates anxiety-like behavior via IL-17a signaling in neurons [55]. These meningeal γδ T cells are virtually absent from the prenatal dural meninges but undergo progressive seeding after birth. However, we found that RORγt is expressed in the brain parenchyma during the embryonic period. In other words, the RORγt-positive cells in the brain parenchyma identified in the present study might not originate from the meninges. However, we were unable to exclude the possibility that *Tsc1* deletion in these meningeal γδ17 T cells would also contribute to brain damage and death, as meningeal γδ17 T cells also express RORγt at high levels. A limitation of the study is that we did not determine the location, type or source of these RORγt-positive cells.

## Conclusions

Taken together, our results verify the critical role of previously unnoticed RORγt-expressing cells in the brain and indicate that the Tsc1 signaling pathway in RORγt-expressing cells is important for maintaining neuron and astrocyte homeostasis.

## Supplementary Information

**Additional file 1: Supplemental Fig. 1.** Abnormal activation of neurons and astrogliosis in the cortex and hippocampus of Ts*c1*^*RORγt*^ mice. a and b Representative images of Nissl staining (a) and GFAP immunostaining (green) (b) in the brains of *Tsc1*^RORγt^ mice and control littermates. All images were captured from scans of whole-brain slices. Magnification: 5x (a and b). Scale bars: 200 μm (a and b).

**Additional file 2: Supplemental Fig. 2.** *RORγt* expression in the mouse brain at different periods. ISH analysis revealed *RORγt* expression during the embryonic period (E18.5) and at PNDs 4, PNDs 14 and PNDs 28. All ISH data were obtained from Allen Brain Map: Developing Mouse Brain Atlas (http://developingmouse.brain-map.org). Image credit: Allen Institute.

**Additional file 3: Supplemental Table 1.** Antibodies used for flow cytometry and immunofluorescence staining.

**Additional file 4: Supplemental Table 2.** Primer pairs used for RT-PCR.

**Additional file 5: Supplemental Table 3.** KEGG analysis of different signal pathway between RORγt-positive cells and RORγt-negative cells.

**Additional file 6: Supplemental Video 1.** Video showing a two-week-old *Tsc1*^*RORγt*^ mouse experiencing spontaneous seizures.

**Additional file 7: Supplemental Video 2.** Video showing a four-week-old *Tsc1*^*RORγt*^ mouse experiencing spontaneous seizures, ultimately resulting in wild jumping and death.

## Data Availability

The authors declare that all data in support of the findings of this study are available within the article and its supplementary information files or from the corresponding author (Dr. Youcai Deng, youcai.deng@tmmu.edu.cn) upon reasonable request.

## Abbreviations

Tsc1: Tuberous sclerosis complex 1
Tsc2: Tuberous sclerosis complex 2
ILCs: Innate lymphoid cells
LTi cells: Lymphoid tissue inducer cells
PND: Postnatal day
LPL: Lamina propria layer
HE: Hematoxylin-eosin
ECG: Electrocardiogram
NeuN: Neuronal nuclei antigen
DG: Dentate gyrus
GFAP: Glial fibrillary acidic protein
GABA: γ-Amino butyric acid
PTZ: Pentylenetetrazole
ISH: In situ hybridization
