# Supplementary figures and images for: Conditional knockout of Tsc1 in RORγt-expressing cells induces brain damage and early death in mice
Source: J Neuroinflammation. 2021 May 6;18:107. doi: 10.1186/s12974-021-02153-8 (PMC8101034; doi:10.1186/s12974-021-02153-8)

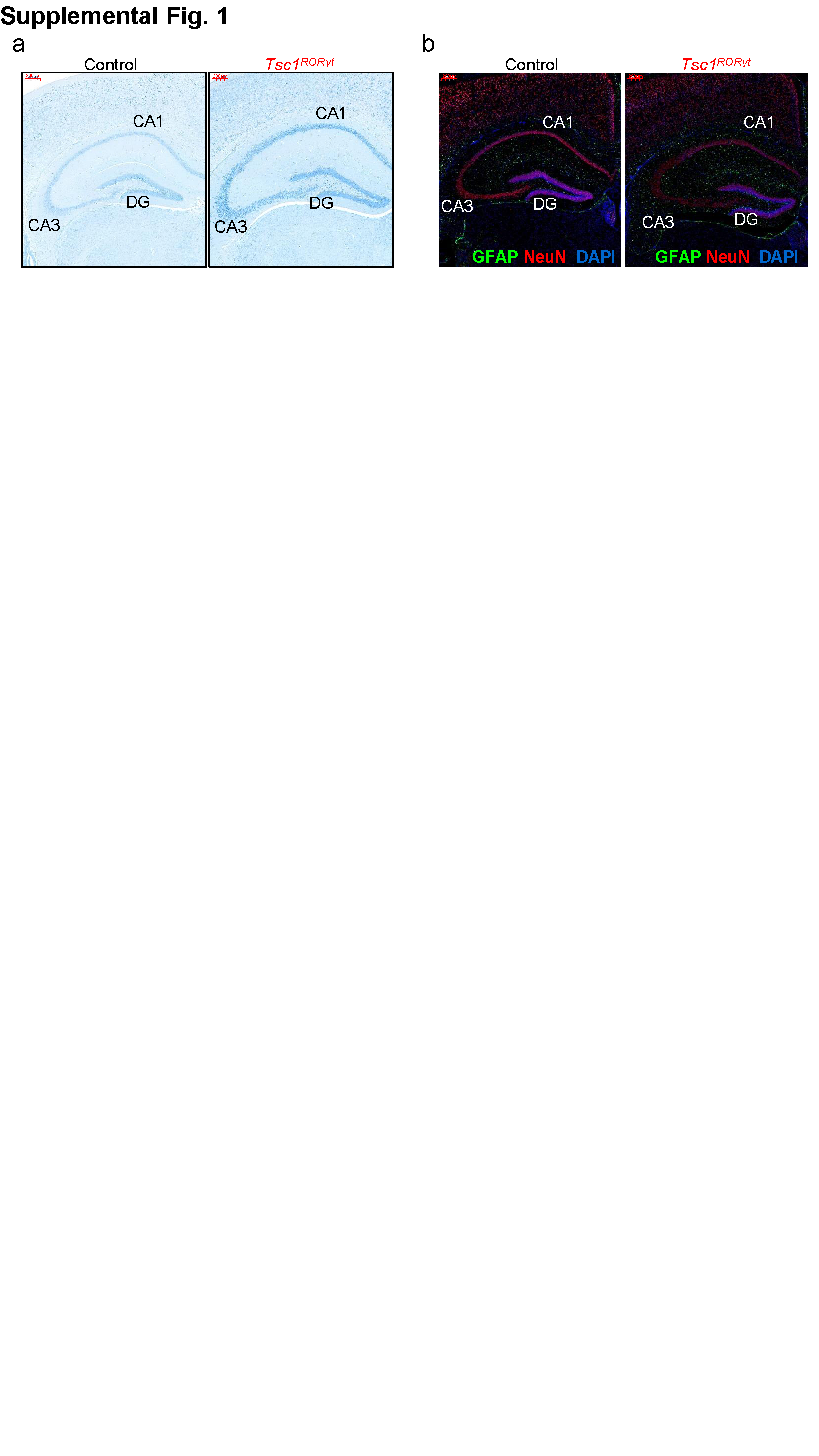

Supplement: Supplementary file 1 — Additional file 1: Supplemental Fig. 1. Abnormal activation of neurons and astrogliosis in the cortex and hippocampus of Tsc1RORγt mice. a and b Representative images of Nissl staining (a) and GFAP immunostaining (green) (b) in the brains of Tsc1RORγt mice and control littermates. All images were captured from scans of whole-brain slices. Magnification: 5x (a and b). Scale bars: 200 μm (a and b). [file 12974_2021_2153_MOESM1_ESM.tiff]

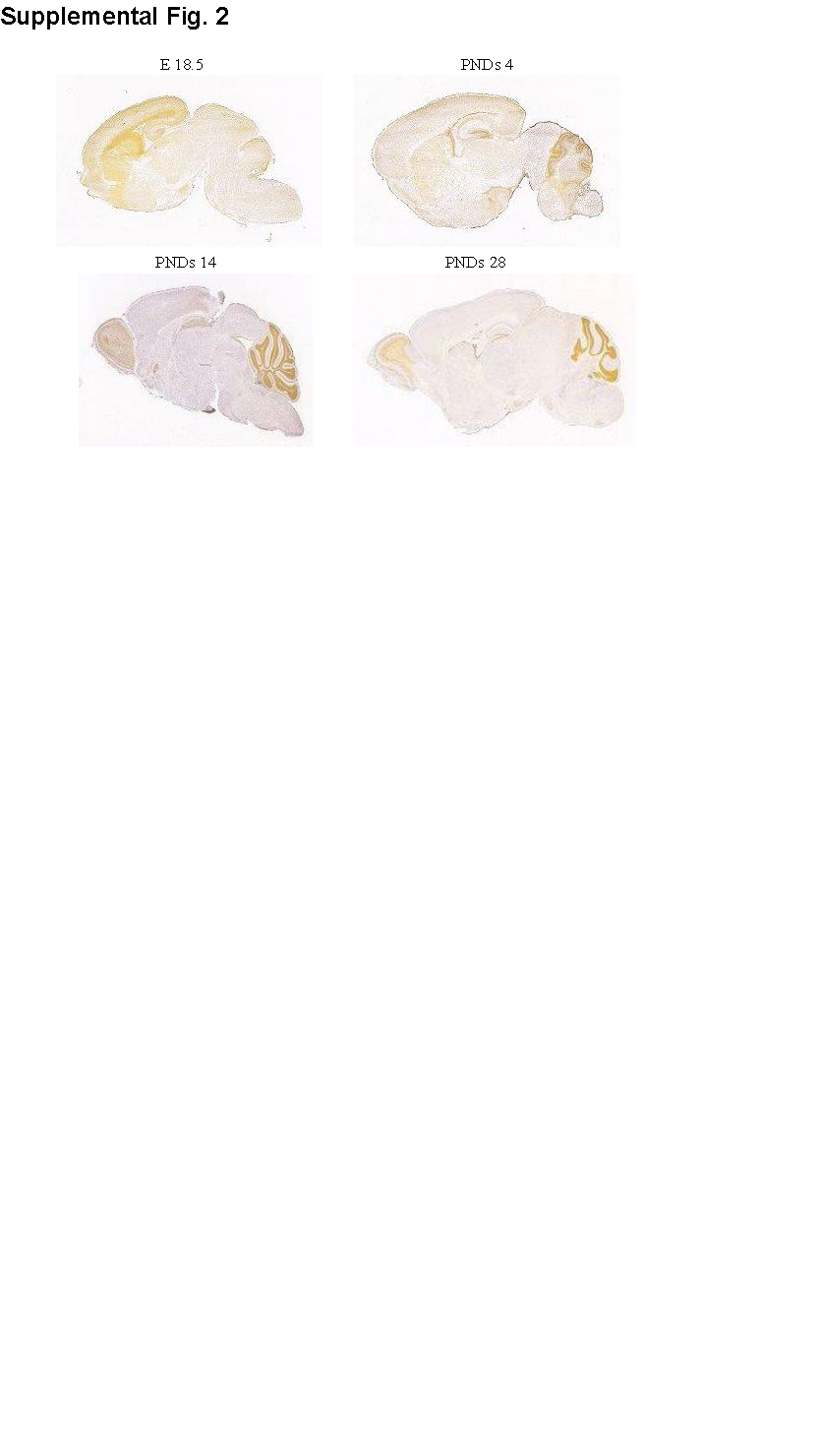

Supplement: Supplementary file 2 — Additional file 2: Supplemental Fig. 2. RORγt expression in the mouse brain at different periods. ISH analysis revealed RORγt expression during the embryonic period (E18.5) and at PNDs 4, PNDs 14 and PNDs 28. All ISH data were obtained from Allen Brain Map: Developing Mouse Brain Atlas (http://developingmouse.brain-map.org). Image credit: Allen Institute. [file 12974_2021_2153_MOESM2_ESM.tiff]
