# Supplementary material for: Conditional knockout of Tsc1 in RORγt-expressing cells induces brain damage and early death in mice
Source: J Neuroinflammation. 2021 May 6;18:107. doi: 10.1186/s12974-021-02153-8 (PMC8101034; doi:10.1186/s12974-021-02153-8)
Supplement: Supplementary file 3 — Additional file 3: Supplemental Table 1. Antibodies used for flow cytometry and immunofluorescence staining. [file 12974_2021_2153_MOESM3_ESM.pdf]

Supplemental Table 1. Antibodies used for flow cytometry and immunofluorescence staining

Antibodies	Clone	Source	Dilution
anti-mouse CD45	30-F11	BioLegend	1/200
anti-mouse CD3e	145-2C11	BioLegend	1/200
anti-mouse CD19	6D5	BioLegend	1/200
anti-mouse B220	RA3-6B2	BioLegend	1/200
anti-mouse Gr-1	RB6-8C5	BioLegend	1/200
anti-mouse CD127	A7R34	BioLegend	1/100
anti-mouse CD90.2	30-H12	BioLegend	1/100
anti-mouse NKp46	29A1.4	BioLegend	1/100
anti-mouse RORγt	Q31-378	BD Biosciences	1/100
anti-mouse CD16/CD32	2.4G2	BD Biosciences	1/100
anti-mouse CD4	RM4-5	BD Biosciences	1/100
anti-mouse CD8a	53-6.7	BD Biosciences	1/100
anti-mouse IL-17A	TC11-18H10	eBioscience	1/50
anti-mouse IL-22	IL22JOP	eBioscience	1/50
anti-GFAP	2.2B10	eBioscience	1/100
anti-NeuN	PA5-78639	eBioscience	1/100
Alexa Fluor® 488 conjugate-goat anti-rat IgG (H+L)	/	eBioscience	1/2000
Alexa Fluor® 594 conjugate-goat anti-rabbit IgG (H+L)	/	eBioscience	1/2000
