# Supplementary material for: Conditional knockout of Tsc1 in RORγt-expressing cells induces brain damage and early death in mice
Source: J Neuroinflammation. 2021 May 6;18:107. doi: 10.1186/s12974-021-02153-8 (PMC8101034; doi:10.1186/s12974-021-02153-8)
Supplement: Supplementary file 4 — Additional file 4: Supplemental Table 2. Primer pairs used for RT-PCR. [file 12974_2021_2153_MOESM4_ESM.pdf]

Supplemental Table 2. Primer pairs used for RT-PCR.

Primer	Forward	Reverse	Classification
rorc	GACCCACACCTCACAAATTGA	AGTAGGCCACATTACACTGCT	RAR-related orphan receptor gamma gene
gabrg1	TGTGGAGTCAAACTAGAGGAGTG	TTCCCAGATGCAGGGTTAGTA	GABA receptor subunit genes
gabra2	GGACCCAGTCAGGTTGGTG	TCCTGGTCTAAGCCGATTATCAT	
gabrb2	ATGTCGCTGGTTAAAGAGACG	CTGCCACTCGGTTGTCCAAA	
gabrb1	TCCCGTGATGGTTGCTATGG	CCGCAAGCGAATGTCATATCC	
gabrb3	CTGCTGCCAATCTGGCTTTC	CGTAGCCTTTCAACAGCTTGTC	
pcdhga2	AAAGCTGGACAGATCCGCTAC	AGCTGCGACTTACCTCTGGA	Neural cadherin-like cell adhesion genes
pcdhga8	CTGGTGCTAGAGCGCACTC	CCGGTTGGTCAAAAACAGGG	
pcdhga9	CGGGCAAATCCGCTATTCC	ACTTCCACCCCATAAAGTTTCAC	
kcnh7	CTTGATACAGCCTAGCCAGTGT	GGTCCTTTGACATTTCGACCATT	Voltage-gated channel genes
kcna3	GGGGCATTGCCATTGTGTC	AGGCGGGATAGTCTTTCTCATC	
scn8a	ATGGGGTAGGCTCTCCGAG	CCGACTCTGACTTAAACACCTTC	
kif5b	GCGGAGTGCAACATCAAAGTG	CATAAGGCTTGGACGCGATCA	Other neuro-functional genes
ube3a	ATCCCAGTCTGAGGACATTGA	GCACAAAACTCATTCGTGCAG	
neto2	GCCCCGGAAAAAGGTTATGG	GCTGTGGTTGTGCAAAATCAT	
erbb4	GTGCTATGGACCCTACGTTAGT	TCATTGAAGTTCATGCAGGCAA	
gapdh	AGGTCGGTGTGAACGGATTTG	TGTAGACCATGTAGTTGAGGTCA	Reference gene
