# Supplementary material for: Conditional knockout of Tsc1 in RORγt-expressing cells induces brain damage and early death in mice
Source: J Neuroinflammation. 2021 May 6;18:107. doi: 10.1186/s12974-021-02153-8 (PMC8101034; doi:10.1186/s12974-021-02153-8)
Supplement: Supplementary file 5 — Additional file 5: Supplemental Table 3. KEGG analysis of different signal pathway between RORγt-positive cells and RORγt-negative cells. [file 12974_2021_2153_MOESM5_ESM.pdf]

Supplemental Table 3. KEGG analysis of different signal pathway between RORγt-positive cells and RORγt-negative cells.

KEGG signal pathway	logFC	P.Value	adj.P.Val
Glycosphingolipid biosynthesis ganglio series	0.457874	1.49E-106	3.94E-105
Glycosaminoglycan biosynthesis heparan sulfate	0.338374	1.90E-90	3.91E-89
Glycosaminoglycan biosynthesis chondroitin sulfate	0.335416	4.53E-84	7.62E-83
Taurine and hypotaurine metabolism	0.325038	4.16E-28	1.92E-27
Glycosaminoglycan degradation	0.31437	1.02E-63	1.11E-62
Other glycan degradation	0.298411	1.01E-43	7.17E-43
Glycerolipid metabolism	0.296011	2.08E-119	7.69E-118
Pantothenate and CoA biosynthesis	0.282096	2.28E-54	2.11E-53
ABC transporters	0.278613	4.68E-91	1.08E-89
Arrhythmogenic right ventricular cardiomyopathy arvc	0.265947	8.32E-121	3.85E-119
β-alanine metabolism	0.259822	3.69E-40	2.28E-39
Alanine aspartate and glutamate metabolism	0.256014	1.05E-53	9.21E-53
Hypertrophic cardiomyopathy hcm	0.25131	3.69E-137	3.41E-135
Amino sugar and nucleotide sugar metabolism	0.25013	7.26E-57	7.07E-56
Glycerophospholipid metabolism	0.247923	8.11E-89	1.50E-87
Dilated cardiomyopathy	0.246595	6.91E-124	4.26E-122
Galactose metabolism	0.231044	2.12E-52	1.71E-51
Glycosaminoglycan biosynthesis keratin sulfate	0.222614	2.48E-21	9.18E-21
ECM receptor interaction	0.222291	6.44E-73	9.17E-72
Glycosylphosphatidylinositol GPI anchor biosynthesis	0.221312	3.63E-29	1.82E-28
Aminoacyl tRNA biosynthesis	0.217898	8.18E-30	4.21E-29
Glycosphingolipid biosynthesis globo series	0.216959	1.13E-27	5.12E-27
Sphingolipid metabolism	0.216104	2.55E-67	3.37E-66
Pentose and glucoronate interconversions	0.211009	1.68E-19	5.56E-19
Inositol phosphate metabolism	0.208903	4.68E-63	4.81E-62
Limonene and pinene degradation	0.208366	4.70E-12	1.11E-11
P53 signaling pathway	0.206363	1.15E-35	6.45E-35
Nitrogen metabolism	0.198616	2.31E-12	5.62E-12
Homologous recombination	0.194647	6.30E-14	1.60E-13
Propanoate metabolism	0.192471	8.34E-20	2.86E-19
Porphyrin and chlorophyll metabolism	0.187092	5.54E-24	2.28E-23
Mismatch repair	0.186459	1.38E-08	2.55E-08
Terpenoid backbone biosynthesis	0.182049	1.95E-08	3.53E-08
Proximal tubule bicarbonate reclamation	0.180862	4.65E-19	1.51E-18
N glycan biosynthesis	0.180243	2.31E-22	8.92E-22
Fructose and mannose metabolism	0.178416	1.49E-24	6.25E-24
Aldosterone regulated sodium reabsorption	0.176625	7.94E-25	3.41E-24

Lysosome	0.170333	5.80E-38	3.46E-37
Type 2 diabetes mellitus	0.168453	4.64E-29	2.26E-28
Basal cell carcinoma	0.160516	1.60E-30	8.73E-30
DNA replication	0.159496	6.93E-06	1.05E-05
Valine leucine and isoleucine degradation	0.154122	1.53E-17	4.78E-17
Nucleotide excision repair	0.151614	2.11E-10	4.48E-10
Adipocytokine signaling pathway	0.148865	1.18E-40	7.53E-40
Circadian rhythm mammal	0.148343	2.28E-07	3.79E-07
mTOR signaling pathway	0.144439	5.70E-17	1.70E-16
Fatty acid metabolism	0.141644	2.49E-14	6.40E-14
RNA degradation	0.140468	2.34E-10	4.92E-10
Lysine degradation	0.140347	6.91E-13	1.73E-12
Progesterone mediated oocyte maturation	0.13923	1.89E-14	4.92E-14
MAPK signaling pathway	0.138602	2.18E-43	1.50E-42
Small cell lung cancer	0.13806	2.44E-27	1.07E-26
Notch signaling pathway	0.137073	1.48E-16	4.34E-16
Non homologous end joining	0.133933	7.12E-11	1.61E-10
Endocytosis	0.133258	5.84E-23	2.35E-22
Folate biosynthesis	0.131713	2.56E-05	3.73E-05
Starch and sucrose metabolism	0.131226	1.16E-22	4.57E-22
Acute myeloid leukemia	0.130776	3.27E-17	9.91E-17
Axon guidance	0.130112	2.36E-16	6.83E-16
Pyrimidine metabolism	0.128321	9.46E-12	2.22E-11
Base excision repair	0.128109	2.10E-07	3.53E-07
Focal adhesion	0.126506	2.18E-28	1.03E-27
RIG-I like receptor signaling pathway	0.126343	7.97E-20	2.78E-19
Calcium signaling pathway	0.124395	1.25E-45	9.22E-45
ERBB signaling pathway	0.124248	1.80E-17	5.55E-17
Protein export	0.123463	0.000145	0.000203
Selenoamino acid metabolism	0.122361	5.75E-11	1.31E-10
Neurotrophin signaling pathway	0.115595	1.36E-10	3.00E-10
Cysteine and methionine metabolism	0.11468	1.05E-07	1.80E-07
Glycine serine and threonine metabolism	0.114673	2.55E-10	5.31E-10
One carbon pool by folate	0.11432	4.11E-07	6.71E-07
Basal transcription factors	0.114282	8.62E-08	1.50E-07
Snare interactions in vesicular transport	0.114023	8.80E-08	1.52E-07
Vascular smooth muscle contraction	0.113328	1.62E-15	4.60E-15
Colorectal cancer	0.110902	1.89E-10	4.06E-10
Amyotrophic lateral sclerosis ALS	0.109006	1.02E-09	2.05E-09
Adherens junction	0.108369	1.88E-10	4.06E-10
Bladder cancer	0.108354	4.81E-08	8.55E-08
Ether lipid metabolism	0.107224	3.48E-09	6.71E-09
Peroxisome	0.106696	1.99E-15	5.51E-15

Pancreatic cancer	0.105903	1.19E-10	2.64E-10
Cell adhesion molecules cams	0.105665	6.88E-22	2.60E-21
Glioma	0.104586	1.33E-08	2.49E-08
Non-small cell lung cancer	0.104171	2.82E-09	5.49E-09
Insulin signaling pathway	0.103682	7.29E-15	1.93E-14
Biosynthesis of unsaturated fatty acids	0.103364	0.000112	0.000158
Chronic myeloid leukemia	0.102708	5.20E-10	1.07E-09
Hedgehog signaling pathway	0.102667	1.99E-15	5.51E-15
Ubiquitin mediated proteolysis	0.101969	4.03E-09	7.70E-09
O-glycan biosynthesis	0.101792	2.14E-15	5.81E-15
Butanoate metabolism	0.098334	2.08E-06	3.23E-06
Fc epsilon RI signaling pathway	0.097573	1.36E-09	2.70E-09
Wnt signaling pathway	0.096712	4.40E-12	1.06E-11
Renal cell carcinoma	0.096529	1.42E-07	2.41E-07
VEGF signaling pathway	0.093701	9.84E-10	2.00E-09
Purine metabolism	0.092804	1.08E-11	2.50E-11
Endometrial cancer	0.092483	6.77E-07	1.09E-06
Leukocyte transendothelial migration	0.091802	7.07E-13	1.74E-12
GNRH signaling pathway	0.091429	4.70E-09	8.87E-09
Long term depression	0.089024	1.05E-06	1.68E-06
Cell cycle	0.088325	5.64E-05	8.09E-05
Cardiac muscle contraction	0.08587	0.000516	0.000691
Long term potentiation	0.083876	8.81E-05	0.000125
Glycosphingolipid biosynthesis lacto and neolacto series	0.083734	1.40E-06	2.19E-06
Toll-like receptor signaling pathway	0.083293	2.70E-15	7.24E-15
Pathways in cancer	0.081149	1.13E-19	3.81E-19
Fc-gamma R mediated phagocytosis	0.080193	1.14E-05	1.69E-05
Cytosolic DNA sensing pathway	0.080012	6.69E-08	1.18E-07
B cell receptor signaling pathway	0.075941	1.26E-06	2.00E-06
Apoptosis	0.074654	2.81E-08	5.05E-08
TGF-β signaling pathway	0.070722	1.89E-08	3.46E-08
Epithelial cell signaling in helicobacter pylori infection	0.068791	0.000302	0.00042
GAP junction	0.067203	0.000414	0.000563
Prostate cancer	0.066487	3.44E-05	4.97E-05
Leishmania infection	0.065798	3.59E-07	5.93E-07
Regulation of actin cytoskeleton	0.063885	2.94E-06	4.53E-06
Tight junction	0.058856	8.14E-06	1.22E-05
Natural killer cell mediated cytotoxicity	0.05693	0.000498	0.000673
Melanogenesis	0.053325	0.000346	0.000474
Arginine and proline metabolism	0.045394	0.000573	0.000757
Neuroactive ligand receptor interaction	0.038378	0.000554	0.000737

JAK-STAT signaling pathway	-0.04482	4.13E-07	6.71E-07
Intestinal immune network for IGA production	-0.05225	0.000313	0.000432
PPAR signaling pathway	-0.05466	3.52E-06	5.39E-06
Viral myocarditis	-0.06465	9.62E-06	1.44E-05
Tyrosine metabolism	-0.07635	2.60E-09	5.11E-09
Primary bileacid biosynthesis	-0.08145	2.32E-05	3.40E-05
Cytokine cytokine receptor interaction	-0.09564	5.30E-19	1.69E-18
Primary immunodeficiency	-0.12638	1.15E-20	4.10E-20
Renin angiotensin system	-0.16523	4.32E-21	1.57E-20
Linoleic acid metabolism	-0.16817	5.34E-30	2.82E-29
Type 1 diabetes mellitus	-0.2052	3.00E-36	1.73E-35
Retinol metabolism	-0.22221	3.28E-66	3.79E-65
Systemic lupus erythematosus	-0.26612	5.16E-49	3.98E-48
Maturity onset diabetes of the young	-0.27808	3.56E-80	5.50E-79
Steroid hormone biosynthesis	-0.31459	1.51E-115	4.64E-114
